# Absences, Symptoms and Respiratory Viruses in a Swiss School: Longitudinal Study With Serial Saliva Sampling

**DOI:** 10.1111/irv.70143

**Published:** 2025-09-29

**Authors:** Nicolas Banholzer, David Kronthaler, Pascal Bittel, Lavinia Furrer, James D Munday, Matthias Egger, Tina Hascher, Philipp Jent, Lukas Fenner

**Affiliations:** ^1^ Institute of Social and Preventive Medicine University of Bern Bern Switzerland; ^2^ Multidisciplinary Center for Infectious Diseases (MCID), University of Bern Bern Switzerland; ^3^ Department of Public Health University of Copenhagen Copenhagen Denmark; ^4^ Institute for Infectious Diseases University of Bern Bern Switzerland; ^5^ Department of Biosystems Science and Engineering ETH Zurich Basel Switzerland; ^6^ Population Health Sciences University of Bristol Bristol UK; ^7^ Centre for Infectious Disease Epidemiology and Research University of Cape Town Cape Town South Africa; ^8^ Department of Infectious Diseases and Hospital Epidemiology University Hospital Zurich, University of Zurich Zurich Switzerland; ^9^ Institute of Educational Science University of Bern Bern Switzerland; ^10^ Department of Infectious Diseases, Inselspital, Bern University Hospital University of Bern Bern Switzerland

**Keywords:** molecular epidemiology, respiratory viruses, school absences, symptoms, transmission

## Abstract

**Background:**

Viral respiratory infections contribute to sick days in school children. We monitored respiratory infections, absences, and symptoms in a Swiss school.

**Methods:**

Serial saliva sampling (three per week) and daily recording of absences and symptoms over 6 weeks during the winter of 2023/24 in four Swiss school classes (age 14–15).

**Results:**

We analyzed 1047 samples of 67/84 (80%) participants, identifying 87 infection episodes across eight viruses: 28 (32%) human rhinovirus, 18 (21%) influenza A/B, 11 (13%) respiratory syncytial virus, 14 (16%) human coronaviruses, 6 (7%) parainfluenza virus, and 5 (6%) influenza B; SARS‐CoV‐2 was not detected. Spatiotemporal trends revealed seasonal epidemic trends and evidence of transmission within classes. Viral loads (interquartile range 29.5–36.9 Ct) and duration of detection (modeled range 3.2–5.3 days) were similar for all viruses. School absences were more likely temporally associated with influenza B infections than with other respiratory viral infections (> 99% vs. 38%, *p* = 0.005), and the absences tended to be longer (average 4.2 vs. 2.2 days). Symptoms varied depending on the pathogen detected, with absences temporally associated with human rhinovirus and parainfluenza virus infections commonly involving runny nose and sore throat, while absences associated with influenza infections often involved fever.

**Conclusions:**

Class‐specific distribution patterns suggest a major contribution of within‐class to overall respiratory virus transmission. Respiratory viruses showed certain distinct profiles in relation to school absences and symptoms. This highlights the importance of infection control measures, including vaccination, and virus‐specific monitoring to better understand transmission dynamics in schools.

## 1 Introduction

Respiratory infections remain a global public health concern, particularly among children and adolescents [1], where they account for a significant proportion of pediatric hospital admissions [2]. Respiratory viral infections spread mainly during the winter months in waves caused by different pathogens occurring simultaneously or consecutively, leading to an annual burden on outpatient and inpatient healthcare [3]. Children and adolescents contribute disproportionately to the spread of respiratory viruses due to frequent close contact and typically lower levels of preexisting immunity [4].

Because children and adolescents spend most of their day in crowded classrooms, schools drive the transmission of respiratory viruses [5]. Respiratory viral infections that cause mild or severe symptoms often lead to school absences [6]. Symptoms and severity vary between viruses [7], and previous studies suggest that the duration and frequency of school absences depend on the virus [8, 9]. Monitoring school absences and respiratory symptoms can indicate levels of viral spread and risk of infection [6], thereby informing strategies to prevent transmission and absences. While there has been extensive research on the prevalence and transmission of respiratory viruses in schools [10, 11, 12], multiple respiratory viruses have rarely been examined over time to investigate their association with school absences and symptoms [13]. Such longitudinal studies with serial sampling are costly, time‐consuming, and require sustained commitment from students and teachers. In contrast, studies using routinely collected data from hospitals and general practitioners can readily support spatiotemporal surveillance but may be biased by omitting mild and asymptomatic infections in individuals who do not seek medical care [2].

We conducted a longitudinal study in four Swiss secondary school classes during the winter of 2023/24, using serial sampling to test for a comprehensive panel of respiratory viruses. We analyzed spatiotemporal trends in viral detection and compared periods of positive molecular tests. We then linked saliva test results to daily recorded school absences to compare periods of absence and symptoms between respiratory viruses.

## 2 Methods

### 2.1 Study Setting

We collected molecular (saliva samples three times a week) and epidemiological (school absences, symptoms) data in four classes (age of students, 14–15 years) of a secondary school in the canton of Bern, Switzerland, for 6 weeks from January 22 to March 8, 2024.

### 2.2 Epidemiological Data

Our study team in the school collected daily data on absences due to illness or other reasons (e.g., accidents, vocational orientation days or unspecified). Neither the students nor the study team were aware of concurring viral infections detected in saliva. For absences due to illness, we asked about 12 common symptoms of respiratory infections: fever, chills, limb pain, loss of taste, loss of smell, fatigue, cough, runny nose, diarrhea, sore throat, headache, shortness of breath, and stomach pains. We classified absences with at least one of these symptoms as illnesses related to respiratory infections. We also recorded such illnesses during the holiday week (February 5–11). Symptoms were typically queried after the student returned to school. If students remained in school, any symptoms they may have experienced were not recorded. Temporal variation of symptoms during illness was also not considered.

### 2.3 Molecular Data

All classes participated in repeated saliva sampling three times per week (Monday, Wednesday, and Friday) using saline saliva collection kits under the guidance of our study team. Samples were transported to the Institute for Infectious Diseases and stored at −80°C until further processing [14]. Saliva samples were analyzed by real‐time PCR using the STARlet IVD/CFX96 Dx/Seegene‐Viewer IVD workflow (Seegene, Seoul, South Korea). We used Seegene's Allplex RV Master Assay, which can detect a panel of 19 major respiratory viruses and viral subtypes, including SARS‐CoV‐2, influenza A/B virus, respiratory syncytial virus (RSV), adenovirus (AdV), human rhinovirus (HRV), and human parainfluenza virus (PIV). We also used the Allplex Respiratory Panel 3, which detects the human coronaviruses NL63, 229E, and OC43.

### 2.4 Linking Viral Infections to School Absences and Definitions

We linked viral infections to coinciding absences if the positive saliva test result was within 1 week of the absence. In some cases, the same viral infection could be attributed to two absences and, vice versa, one absence could be attributed to two viral infections. Co‐infections (simultaneous detection of more than one pathogen) were also attributed to the same absence(s). Periods of positive molecular viral tests (duration of detection) were defined as the number of consecutive days with positive saliva test results. Periods were separated if more than 1 week elapsed between two positive saliva test results. The lowest cycling time (Ct) value per infection episode was used as a semiquantitative measure of peak viral load for statistical comparison.

### 2.5 Statistical Analysis

Continuous variables are reported as medians (interquartile range [IQR]) and categorical variables as counts (proportion [%]). Differences in viral loads (Ct values) between viruses were estimated using linear regression with post hoc pairwise comparisons, reporting the mean differences (95%‐confidence interval [CI]) and *p*‐values from F‐tests. Class and sex differences in virus detection were analyzed using Fisher's exact test. The duration of viral detection in saliva was modeled using proportional hazard models, adjusting for school‐free days and absences. The proportion of viral infections coinciding with an absence was modeled with a binomial distribution. For absences attributed to viral infections, the duration of the virus‐specific absence period was also estimated using a proportional hazard model, adjusting for school‐free days. Estimation results were reported as means or medians with 95%‐CIs, and differences between viruses were reported with risk differences, hazard or odds ratios, and *p*‐values. Finally, we compared the frequency of 12 self‐reported symptoms and analyzed similarity between respiratory viruses using hierarchical cluster analysis. Statistical analyses were performed using R version 4.3.1.

### 2.6 Ethical Statement

The Ethics Committee of the Canton of Bern, Switzerland, approved the study (reference no. 2023‐02035). All students willing to participate in the saliva sampling were included, with written informed consent obtained from students and their caregivers.

## 3 Results

### 3.1 Student Characteristics and Respiratory Viruses Detected

**FIGURE 1:**
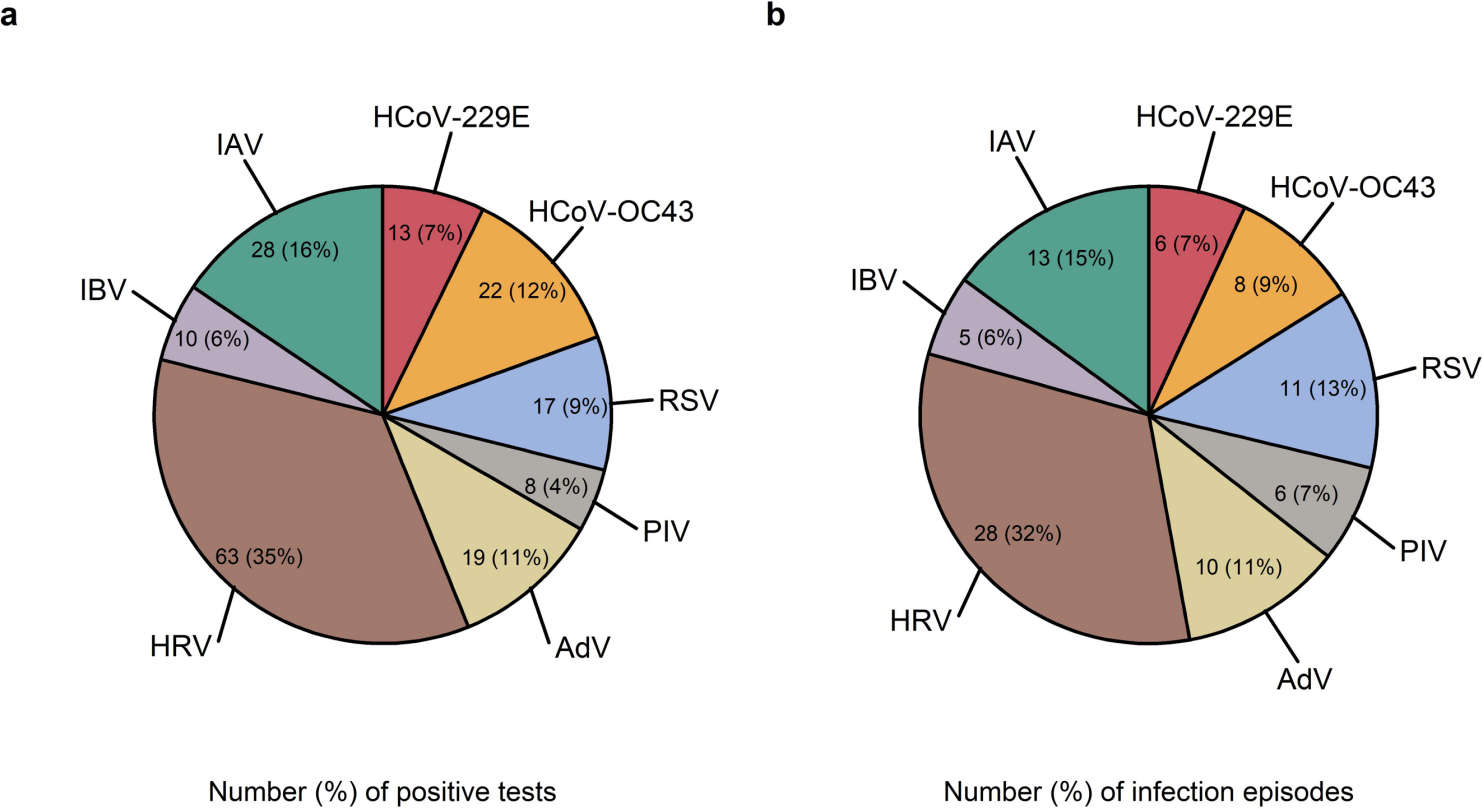
Respiratory viruses detected in student saliva. (a) Number and proportion of positive saliva tests per pathogen (*N* = 180) and (b) number and proportion of infections per pathogen (*N* = 87). IAV: influenza A; IBV: influenza B; HRV: human rhinovirus; AdV: adenovirus; PIV: human parainfluenza virus; RSV: respiratory syncytial virus; HCoV‐OC43: human coronavirus OC43; HCoV‐229E: human coronavirus 229E.

Of 84 students, 67 (80%) participated in serial saliva testing (Table 1). We analyzed 1047 samples, of which 180 (17%) tested positive for any respiratory virus (Figure 1a). During the study, 52 (78%) students had at least one infection, with a total of 87 infection episodes (Figure 1b): 28 (32%) HRV, 13 (15%) influenza A virus (IAV), 11 (13%) RSV, 10 (11%) AdV, 8 (9%) human coronavirus OC43 (HCoV‐OC43), 6 (7%) human coronavirus 229E (HCoV‐229E), 6 (7%) PIV, and 5 (6%) influenza B virus (IBV). SARS‐CoV‐2 was not detected. We identified seven co‐infections with two (2x HRV & HCoV‐43, 1x HRV & PIV, 1x AdV & HRV, 1x AdV & IAV, 1x HCoV‐OC43 & IAV, and 1x IAV & RSV) and one co‐infection with three (IAV & AdV & RSV) different viruses. The median of the lowest Ct value per viral infection was 31.2 (IQR 28.8–35.5) for RSV and 31.4 (IQR 29.8–33.5) for IAV, followed by 32.7 (IQR 28.1–36.1) for IBV, 33.4 (IQR 30.9–35.8) for HCoV‐OC43, 35.4 (IQR 29.3–37.2) for HRV, 35.7 (IQR 33.1–37.7) for AdV, 35.8 (IQR 33.0–37.9) for HCoV‐229E, and 36.8 (IQR 35.5–37.5) for PIV. Differences in viral loads (Ct values) were not statistically significant between respiratory viruses (*p*‐value: 0.40, see also Figure S1), nor between male and female students (*p* = 0.48).

**TABLE 1:** Overview of the study population, number of absences, and number of positive saliva samples and positively tested students over the study.

	Overall	Class 1	Class 2	Class 3	Class 4
	*n* (%)	*n* (%)	*n* (%)	*n* (%)	*n* (%)
Participants	67/84 (79.8)	18/18 (100)	17/24 (70.8)	15/18 (83.3)	17/24 (70.8)
Sex					
Male	41 (61.2)	12 (66.7)	8 (47.1)	9 (60.0)	12 (70.6)
Female	26 (38.8)	6 (33.3)	9 (52.9)	6 (40.0)	5 (29.4)
Absences	105	29	21	27	28
Respiratory disease	81 (77.1)	22 (75.9)	14 (66.7)	22 (81.5)	23 (82.1)
Other illnesses	4 (3.8)	1 (3.4)	1 (4.8)	1 (3.7)	1 (3.6)
Unrelated to illness	20 (19.0)	6 (20.7)	6 (28.6)	4 (14.8)	4 (14.3)
Positive saliva tests	180/1047 (17)	29/299 (9.7)	73/281 (25.9)	28/244 (11.5)	50/223 (21.5)
Students with infection	52/67 (77.6)	13/18 (72.2)	14/17 (82.4)	10/15 (66.7)	15/17 (88.2)
Infection episodes	87	15	30	18	24

### 3.2 Spatiotemporal Distribution of Respiratory Viruses

**FIGURE 2:**
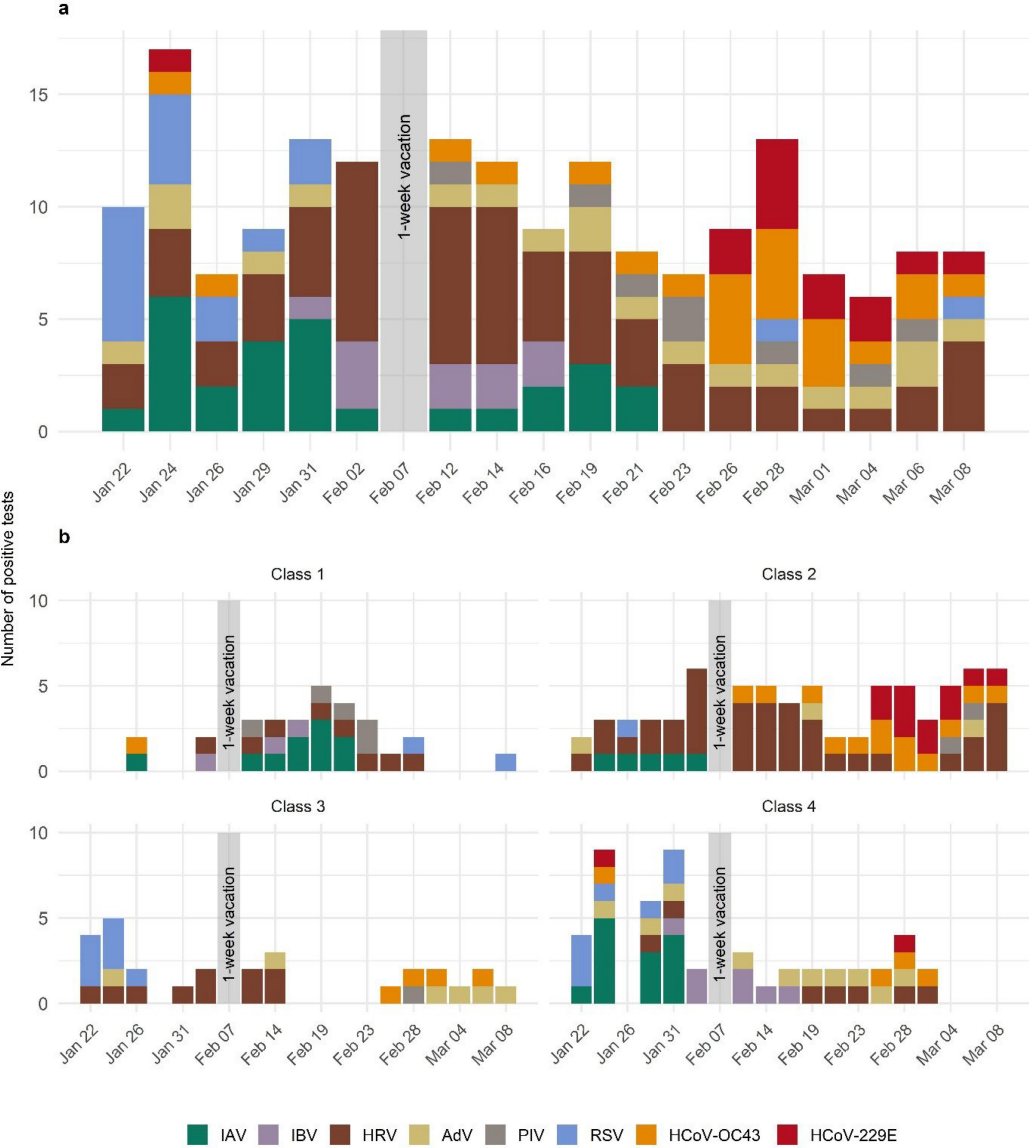
Temporal distribution of positive saliva samples. Number of positive saliva samples over time: (a) overall and (b) by class. IAV: influenza A; IBV: influenza B; HRV: human rhinovirus; AdV: adenovirus; PIV: human parainfluenza virus; RSV: respiratory syncytial virus; HCoV‐OC43: human coronavirus OC43; HCoV‐229E: human coronavirus 229E.

The temporal distribution of positive saliva samples varied over time (Figure 2). HRV was detected throughout the study; RSV was mainly observed at the start in January, IAV was only detected in the first half until February, and seasonal coronaviruses were mainly detected at the end. Detection rates varied between classes (*p* < 0.001), suggesting within‐classroom transmission. IAV was not detected in Class 3 and was only detected in Class 1 after the holidays. HRV was primarily found in Class 2, IBV mainly in Class 4, and HCoV‐229E predominantly in Class 2. Detection rates of different respiratory viruses did not differ by sex (*p* = 0.52), with only RSV and IBV tending to be more common in male and HCoV‐229E in female students (Figure S2); PIV was not detected in female students.

### 3.3 Duration of Detection of Respiratory Viruses and Absences From School

**FIGURE 3:**
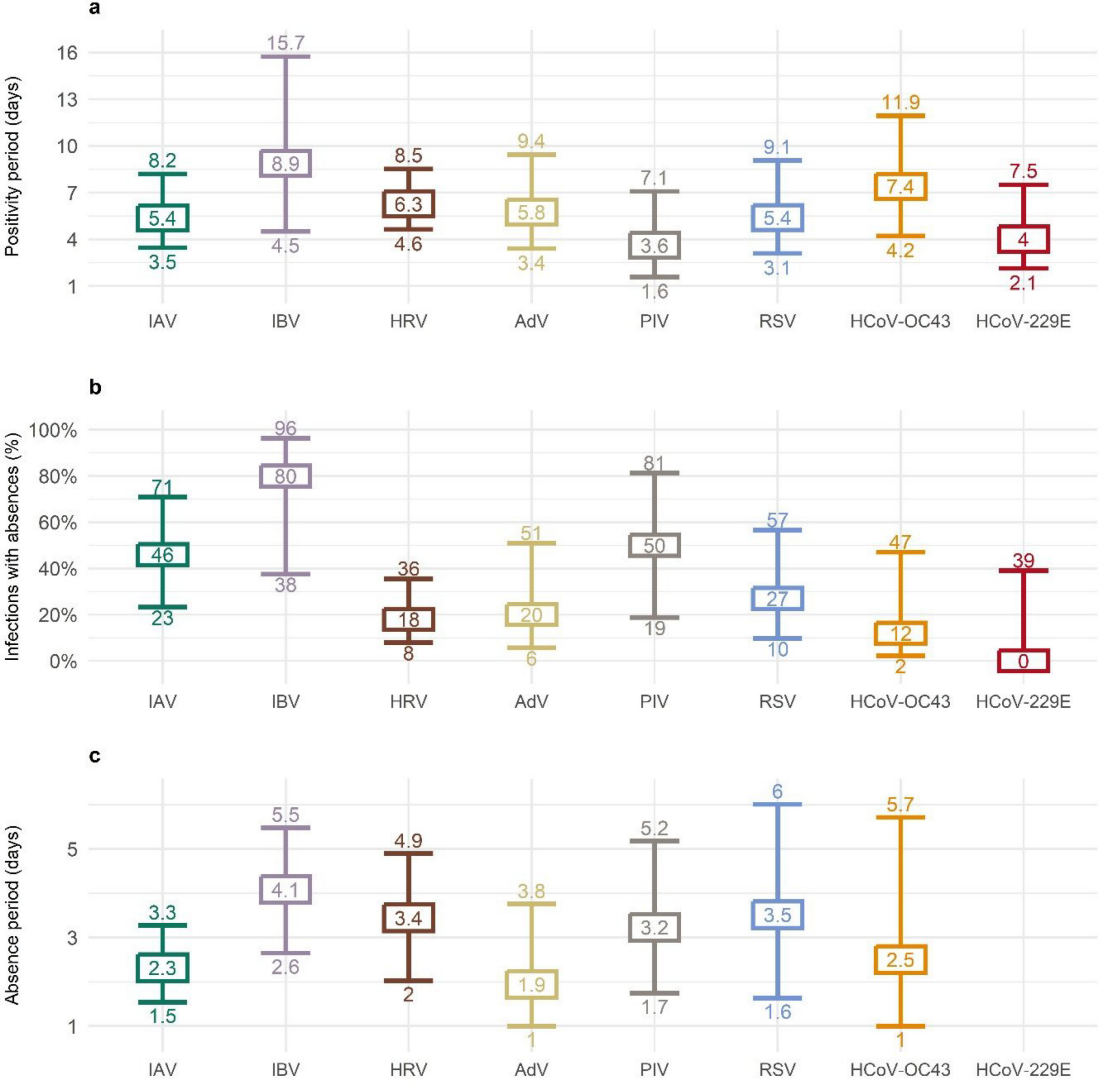
Duration of viral detection in saliva and school absence period by respiratory virus. Shown are the estimates and 95%‐CIs for the (a) median number of days testing positive, (b) the mean proportion of infection episodes temporally associated with an absence with at least one of 12 symptoms of respiratory infections, and (c) the median number of days absent from school for viral infections linked to such absences. HCoV‐229E infection was never linked to an absence. IAV: influenza A; IBV: influenza B; HRV: human rhinovirus; AdV: adenovirus; PIV: human parainfluenza virus; RSV: respiratory syncytial virus; HCoV‐OC43: human coronavirus OC43; HCoV‐229E: human coronavirus 229E.

The duration of detection was similar for all respiratory viruses (median 4.1 days, 95%CI 3.2–5.3, Figure 3a and Table S1; Figures S3 and S4 for detailed comparisons). Of 87 infections detected in saliva, 37 (43%) occurred within 1 week of absences with reported symptoms of respiratory infections (Figure 3b); the other 50 (57%) viral infections were not temporally associated with absences. Vice versa, 45 (55%) of absences with reported symptoms of respiratory infections did not occur within 1 week that a viral infection was detected. IBV infections were more frequently associated with absences from school (proportion > 99%, 95%‐CI 61%–100%), more so than other respiratory viral infections (38%, 95%‐CI 28%–49%, *p* = 0.005, see Figure S5 for pairwise risk differences). Absences temporally associated with viral infections (median 2.3 days, 95%‐CI 1.6–3.2) were about 1 day longer than absences not temporally associated with viral infections (median 1.6 days, 95%‐CI 1.2–2.2; hazard ratio 0.70, *p* = 0.1). With an average length of 4.2 days (95%‐CI 2.1–6.7), absences within 1 week of detected IBV infections tended to be almost two times longer than those within 1 week of other detected respiratory viral infections (2.2, 95%‐CI 1.5–3.0, hazard ratio 0.54, *p* = 0.12, Figure 3c; see Table S2 and Figures S6 and S7 for detailed comparisons).

### 3.4 Symptoms of Respiratory Virus

**FIGURE 4:**
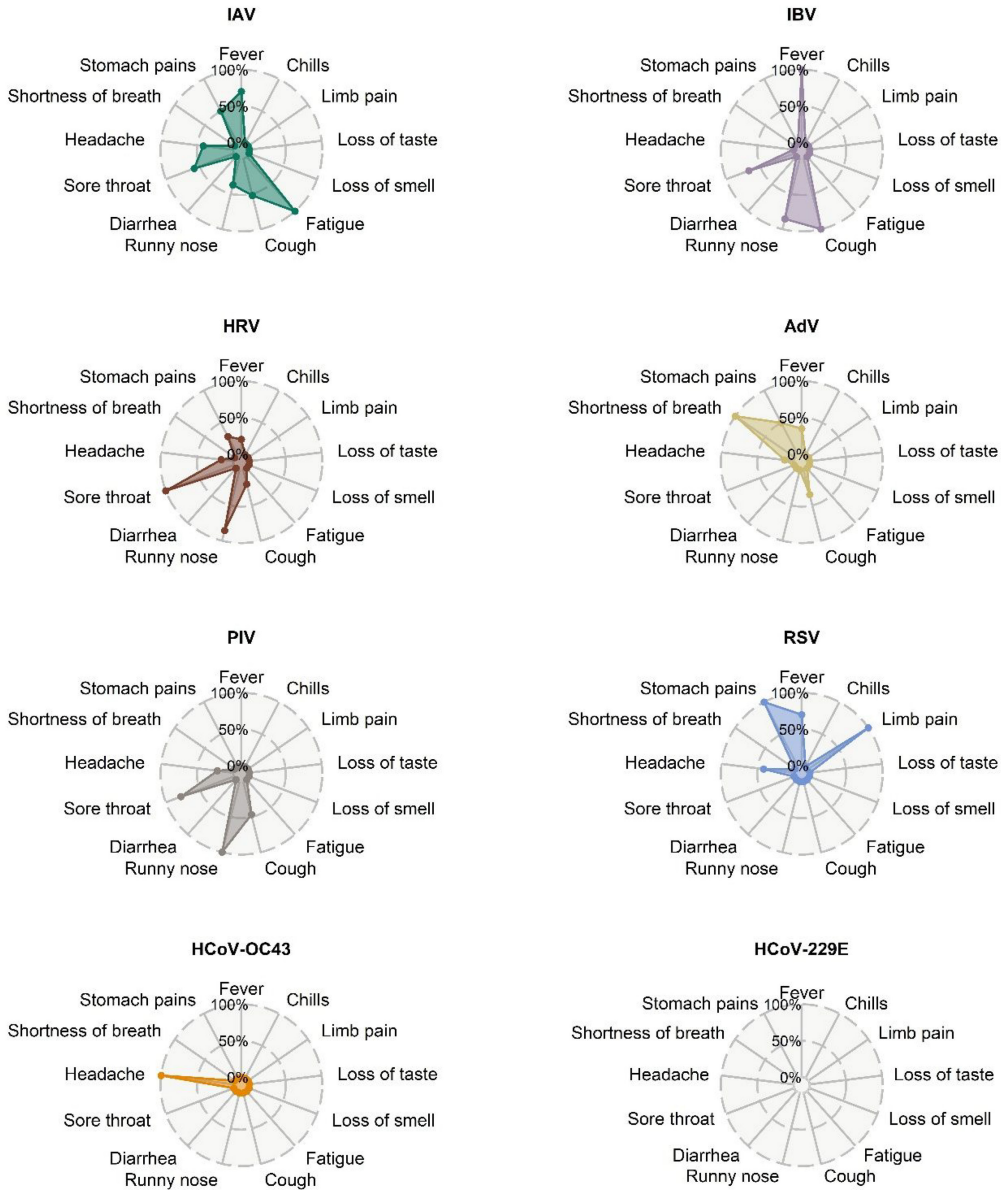
Distribution of symptoms by respiratory virus. Based on absences attributed to viral infections detected in saliva. Proportion of absences as length of spokes. IAV: influenza A; IBV: influenza B; HRV: human rhinovirus; AdV: adenovirus; PIV: human parainfluenza virus; RSV: respiratory syncytial virus; HCoV‐OC43: human coronavirus OC43; HCoV‐229E: human coronavirus 229E.

There were similarities in respiratory symptoms (Figure 4). Common symptoms reported for absences within 1 week of detected HRV and PIV infections included runny nose (55% and 67% of absences, respectively) and sore throat (100% and 61% of absences, respectively). Students with absences within 1 week of detected IAV and IBV infections commonly reported fever (62% and 100% of absences, respectively). Hierarchical cluster analysis confirmed these similarities (Figure S8). Symptoms reported for absences within 1 week of detected viral infections were similar when excluding co‐infections linked to the same absence or when allowing 4 or 10 days to elapse between an absence and a detected viral infection, except for some symptoms of RSV and HCoV‐OC43 (Figure S9 and S10). For absences within 1 week of detected RSV infections, stomach pains, runny nose, loss of taste, and headaches remained common.

## 4 Discussion

We monitored respiratory viral infections in student saliva and tracked school absences over a six‐week period during the winter of 2023/24 in four classes of a Swiss school. Several respiratory viruses were detected in the saliva samples, including HRV, influenza A/B, AdV, RSV, and seasonal common coronaviruses, although notably, we did not detect SARS‐CoV‐2. A pattern emerged showing that respiratory pathogens were specific to certain classrooms and periods, but not to sex. We identified virus‐specific trends in the likelihood of infection coinciding with absence, the length of absence, and the symptoms reported. Absences from school were more likely for influenza B infections and tended to be longer. Influenza A and B infections were often associated with fever and differed from the symptom profiles of other respiratory viruses. Duration of detection and viral load in saliva were similar for all viruses.

A strength of our study is the serial saliva sampling in a large sample of multiple school classes, which allowed the identification of temporal trends and spatial patterns. Spatiotemporal analysis of positive saliva test results suggested transmission within classes, consistent with intra‐classroom spread [15, 16]. Students of the same class spend more time indoors and in close contact, likely facilitating the transmission of respiratory infections [17, 18]. Signs of transmission within schools and classrooms are supported by prior research showing frequent contamination of desk surfaces in schools with respiratory viruses [19]. Although contact rates may vary by sex, we did not observe statistically significant differences in the virus‐specific detection rates by sex. However, our study may have been underpowered to detect such differences. A longitudinal study following symptomatic primary school children in rural Kenya found a higher proportion of virus‐positive nasopharyngeal swabs among males [20]. A study in India also found sex differences in children and adolescents, with males experiencing more frequent upper respiratory tract infections than females [21].

Temporal analysis of viral infections revealed several trends. Most infections with influenza and RSV were detected until early February. Previous studies have also found that RSV in the temperate northern hemisphere often peaks in early winter, while the peak for influenza may vary by season, but generally falls between January and late February [22]. In contrast, seasonal human coronaviruses OC43 and 229E were primarily detected in March. Other respiratory viruses, particularly HRV, were observed throughout the study period, suggesting a lack of strong seasonality. SARS‐CoV‐2 was not detected during our six‐week study in the winter of 2023/2024, rarely during our seven‐week study in 2022/2023 [15], and almost exclusively in the winter of 2021/2022 during the Omicron wave [23]. Other studies have also noted this shift from predominantly SARS‐CoV‐2 during the pandemic to primarily non‐SARS‐CoV‐2 infections in recent years, including influenza viruses and RSV [24, 25]. These temporal trends and peaks in the post‐pandemic era are largely reflected in the epidemiology of respiratory viruses in the general population in Europe [26].

Simultaneous infections with multiple viruses were rare in our study. Low co‐infection rates were also reported in studies using medical center convenience sampling [27, 28]. In contrast, a study at a tertiary pediatric hospital frequently detected co‐infections in patients with clinically significant respiratory symptoms [29]. These discrepancies likely stem from differences in study settings and data collection methods. We used saliva samples, which typically have lower viral loads (higher Ct values) than the nasopharyngeal swab samples used in the hospital study [14]. Hospitalized patients are predominantly symptomatic children, while our school‐based study also captured respiratory infections in asymptomatic children. Most infections detected in saliva were not linked to absences, suggesting that many students experienced no, or only mild, symptoms, allowing them to remain in school. However, asymptomatic students may still contribute to transmission. The US Centers for Disease Control and other public health agencies recommend routine control measures such as improving air quality, promoting hand hygiene, and vaccination [30]. School‐based influenza vaccination programs have shown reductions in influenza‐like illnesses, school absences, and missed workdays among adults in the same household [31]. In the UK, the national vaccination program was extended to include healthy children and adolescents, showing both direct and indirect protection [32]. However, these studies primarily used intranasal live attenuated influenza vaccines, which are not available in many European countries.

IAB infections were more likely associated with an absence and with longer absence periods, suggesting that these infections were presenting with more severe symptoms than other respiratory infections. More severe symptoms have generally been observed for influenza infections [33], but previous studies have found the clinical severity of IAV infections to be higher [34] or comparable [35] to the severity of IAB infections. Infections with HRV, RSV, parainfluenza virus, and seasonal coronaviruses were less frequently associated with absences. When they occurred, they tended to be shorter than absences associated with influenza B infections, consistent with previous findings [8].

The distribution of reported symptoms revealed distinct, virus‐specific profiles that generally align with previous findings. For example, students infected with influenza frequently reported fever [11], in contrast to those infected with seasonal coronaviruses, who were less likely to be absent or mainly reported headaches. Symptoms of RSV involved loss of taste and stomach pain, which differed from cough and shortness of breath reported in a study with young and adult patients in primary care [11]. These discrepancies may be due to the setting (school vs. healthcare facilities) or age. Additionally, the sample size for certain viruses in our study was small because many infections were not linked to absences. Specifically, infections with RSV were found primarily at the beginning and may have been associated with absences that occurred before the study started.

Our study has several limitations. First, despite high participation and serial saliva sampling three times per week, some viral infections may have been missed in non‐participating students or if students became absent before test results could be obtained. We found that 55% of the absences with symptoms of respiratory infections did not occur within 1 week that a viral infection was detected in saliva. Conversely, some viral infections may not have led to absences, yet students could still have experienced symptoms. We found that 57% of viral infections were not detected within 1 week of absences with symptoms of respiratory infections and note that possible symptoms for these infections in school were not recorded. The severity of symptoms was also not recorded. Second, viral infections linked to absences may not mean that the infection caused the symptoms associated with the absence. Hence, our findings should be interpreted as associative rather than causal links. Third, the symptoms recorded in the context of absences may be subject to bias if students make up symptoms to justify their absence or are unable to recall them. To minimize such biases, symptoms were reported in private without the teachers' knowledge. Fourth, our study population consisted of students aged 14–15 years and our findings may not generalize to other age groups. We could also only enroll a limited number of students, impeding statistical comparison, especially for viruses rarely detected in our study. Fifth, while our molecular assays have not been formally validated for respiratory viruses other than SARS‐CoV‐2 in saliva, this sample type is increasingly preferred over nasopharyngeal swabs for monitoring respiratory infections due to easier collection and potentially similar accuracy [36]. Finally, our six‐week period may not be long enough to reveal the entire seasonal epidemiology of respiratory viruses. However, the study period covered the main season of respiratory transmission in winter. In addition, our serial saliva sampling was intensive (three times per week), allowing a detailed longitudinal analysis of respiratory virus circulation in multiple school classes and the association with absences and symptoms.

In conclusion, this study provides a comprehensive view of respiratory virus spread, highlighting the range of pathogens circulating among students during winter. It demonstrates the effectiveness of serial saliva collection for tracking respiratory viral transmission in schools. Different viral circulation between school classes suggests that within‐class transmission is an important contributor to overall respiratory virus transmission. By linking molecular test results with school absences, we also provide a clearer understanding of which viral infections are likely to lead to absences and what symptoms are associated with different viruses. These findings emphasize the importance of virus‐specific monitoring to better understand transmission dynamics in schools, as well as the importance of simple infection control measures, including vaccination. Future studies should explore the role of asymptomatic carriers in virus spread and include behavioral data, such as social contact networks, and environmental factors, such as ventilation and classroom occupancy, in educational settings.

## Author Contributions

**Nicolas Banholzer:** conceptualization, data curation, formal analysis, visualization, writing – review and editing, writing – original draft, investigation, project administration, validation. **David Kronthaler:** validation, visualization, writing – review and editing, writing – original draft, formal analysis, data curation. **Pascal Bittel:** conceptualization, investigation, funding acquisition, writing – review and editing, methodology. **Lavinia Furrer:** investigation, writing – review and editing, methodology, data curation. **James Munday:** investigation, writing – review and editing. **Matthias Egger:** resources, writing – review and editing, writing – original draft, funding acquisition. **Tina Hascher:** writing – review and editing, conceptualization, funding acquisition, investigation. **Philipp Jent:** funding acquisition, writing – review and editing, investigation, conceptualization, resources. **Lukas Fenner:** conceptualization, investigation, funding acquisition, writing – original draft, writing – review and editing, project administration, supervision, resources.

## Conflicts of Interest

The authors declare no conflicts of interest.

## Supporting information


**Table S1:** Comparison of the duration of detection. Hazard ratio that each pathogen's duration is longer than that of HCoV‐229E (shortest period). IAV: influenza A; IBV: influenza B; HRV: human rhinovirus; AdV: adenovirus; PIV: human parainfluenza virus; RSV: respiratory syncytial virus; HCoV‐OC43: human coronavirus OC43; HCoV‐229E: human coronavirus 229E.
**Table S2:** Comparison of school absence periods. Hazard ratio that each pathogen's period is longer than that of RSV (shortest period). No comparison with HCoV‐229E because no coinciding absences recorded. IAV: influenza A; IBV: influenza B; HRV: human rhinovirus; AdV: adenovirus; PIV: human parainfluenza virus; RSV: respiratory syncytial virus; HCoV‐OC43: human coronavirus OC43; HCoV‐229E: human coronavirus 229E.
**Figure S1:** Comparison of viral loads. (a) Viral loads as the median and interquartile range (IQR) of the lowest Ct values per infection episode. (b) Estimated differences in mean Ct values: *p* < 0.05 (*), *p* < 0.01 (**), *p* < 0.001 (***). IAV: influenza A; IBV: influenza B; HRV: human rhinovirus; AdV: adenovirus; PIV: human parainfluenza virus; RSV: respiratory syncytial virus; HCoV‐OC43: human coronavirus OC43; HCoV‐229E: human coronavirus 229E.
**Figure S2:** Temporal distribution of positive saliva samples by sex. Number of positive saliva samples over time by sex. IAV: influenza A; IBV: influenza B; HRV: human rhinovirus; AdV: adenovirus; PIV: human parainfluenza virus; RSV: respiratory syncytial virus; HCoV‐OC43: human coronavirus OC43; HCoV‐229E: human coronavirus 229E.
**Figure S3:** Duration of detection of respiratory viruses in saliva. Observed duration of detection as the number of days testing positive. IAV: influenza A; IBV: influenza B; HRV: human rhinovirus; AdV: adenovirus; PIV: human parainfluenza virus; RSV: respiratory syncytial virus; HCoV‐OC43: human coronavirus OC43; HCoV‐229E: human coronavirus 229E.
**Figure S4:** Virus‐specific duration of detection as survival functions. Estimated probability of testing positive as function of the number of days since the first positive saliva test result. IAV: influenza A; IBV: influenza B; HRV: human rhinovirus; AdV: adenovirus; PIV: human parainfluenza virus; RSV: respiratory syncytial virus; HCoV‐OC43: human coronavirus OC43; HCoV‐229E: human coronavirus 229E.
**Figure S5:** Comparison of the frequencies of viral infections involving school absences. Risk differences (percentage points) for the proportion of school absences with symptoms of respiratory infections linked to viral infections detected in saliva. IAV: influenza A; IBV: influenza B; HRV: human rhinovirus; AdV: adenovirus; PIV: human parainfluenza virus; RSV: respiratory syncytial virus; HCoV‐OC43: human coronavirus OC43; HCoV‐229E: human coronavirus 229E.
**Figure S6:** Duration of school absence periods attributed to respiratory viruses. Observed school absence as the number of days being absent from school due to an illness with symptoms of respiratory infections. IAV: influenza A; IBV: influenza B; HRV: human rhinovirus; AdV: adenovirus; PIV: human parainfluenza virus; RSV: respiratory syncytial virus; HCoV‐OC43: human coronavirus OC43; HCoV‐229E: human coronavirus 229E.
**Figure S7:** Virus‐specific absence period as survival functions. Estimated probability of being absent as function of the number of days since the start of the absence. IAV: influenza A; IBV: influenza B; HRV: human rhinovirus; AdV: adenovirus; PIV: human parainfluenza virus; RSV: respiratory syncytial virus; HCoV‐OC43: human coronavirus OC43; HCoV‐229E: human coronavirus 229E.
**Figure S8:** Clustering of respiratory viruses with common symptoms. Hierarchical cluster analysis showing the cophenetic distance between respiratory viruses based on recorded symptoms. Viruses with common symptoms have a lower distance and are connected closer to the roots of the cluster tree. HCoV‐229E not shown because no coinciding absences and symptoms recorded. IAV: influenza A; IBV: influenza B; HRV: human rhinovirus; AdV: adenovirus; PIV: human parainfluenza virus; RSV: respiratory syncytial virus; HCoV‐OC43: human coronavirus OC43; HCoV‐229E: human coronavirus 229E.
**Figure S9:** Sensitivity analysis for the distribution of symptoms by respiratory virus when excluding absences linked to co‐infections. Based on absences attributed to viral infections detected in saliva. Proportion of absences as length of spokes. IAV: influenza A; IBV: influenza B; HRV: human rhinovirus; AdV: adenovirus; PIV: human parainfluenza virus; RSV: respiratory syncytial virus; HCoV‐OC43: human coronavirus OC43; HCoV‐229E: human coronavirus 229E.
**Figure S10:** Sensitivity analysis for the distribution of symptoms by respiratory virus when linking absences 4, 7 and 10 days before or after a viral infection. Based on absences attributed to viral infections detected in saliva. Proportion of absences as length of spokes. IAV: influenza A; IBV: influenza B; HRV: human rhinovirus; AdV: adenovirus; PIV: human parainfluenza virus; RSV: respiratory syncytial virus; HCoV‐OC43: human coronavirus OC43; HCoV‐229E: human coronavirus 229E.

## Data Availability

Code is available from https://github.com/davidkronthaler‐dk/mcid3_absences_symptoms. Data are available upon reasonable request to the corresponding author.
