# Supplementary material for: Absences, Symptoms and Respiratory Viruses in a Swiss School: Longitudinal Study With Serial Saliva Sampling
Source: Influenza Other Respir Viruses. 2025 Sep 29;19(10):e70143. doi: 10.1111/irv.70143 (PMC12477406; doi:10.1111/irv.70143)

**SUPPLEMENTARY INFORMATION**

Supplementary Table 1: Comparison of the duration of detection. Hazard ratio that each pathogen’s duration is longer than that of HCoV-229E (shortest period). IAV: influenza A; IBV: influenza B; HRV: human rhinovirus; AdV: adenovirus; PIV: human parainfluenza virus; RSV: respiratory syncytial virus; HCoV-OC43: human coronavirus OC43; HCoV-229E: human coronavirus 229E.

|  | **Hazard ratio** | **Lower CI (95%)** | **Upper CI (95%)** | ***p*-value** |
| --- | --- | --- | --- | --- |
| IAV | 0.62 | 0.23 | 1.66 | 0.34 |
| IBV | 0.64 | 0.18 | 2.25 | 0.48 |
| HRV | 0.63 | 0.26 | 1.58 | 0.33 |
| AdV | 0.71 | 0.25 | 2.02 | 0.52 |
| PIV | 0.66 | 0.18 | 2.35 | 0.52 |
| RSV | 0.93 | 0.34 | 2.58 | 0.90 |
| HCoV-OC43 | 0.48 | 0.16 | 1.44 | 0.19 |
| HCoV-229E (reference) | 1 | - | - | - |

Supplementary Table 2: Comparison of school absence periods. Hazard ratio that each pathogen’s period is longer than that of RSV (shortest period). No comparison with HCoV-229E because no coinciding absences recorded. IAV: influenza A; IBV: influenza B; HRV: human rhinovirus; AdV: adenovirus; PIV: human parainfluenza virus; RSV: respiratory syncytial virus; HCoV-OC43: human coronavirus OC43; HCoV-229E: human coronavirus 229E.

|  | **Hazard ratio** | **Lower CI (95%)** | **Upper CI (95%)** | ***p*-value** |
| --- | --- | --- | --- | --- |
| IAV | 0.96 | 0.29 | 3.19 | 0.95 |
| IBV | 0.29 | 0.08 | 1.04 | 0.06 |
| HRV | 0.41 | 0.12 | 1.39 | 0.15 |
| AdV | 0.38 | 0.09 | 1.59 | 0.19 |
| PIV | 0.63 | 0.14 | 2.92 | 0.56 |
| RSV (reference) | - | - | - | - |
| HCoV-OC43 | 0.81 | 0.14 | 4.73 | 0.81 |

**Supplementary Figure 1:** **Comparison of viral loads.** **(a)** Viral loads as the median and interquartile range (IQR) of the lowest Ct values per infection episode. **(b)** Estimated differences in mean Ct values: *p* < 0.05 (*), *p* < 0.01 (**), *p* < 0.001 (***). IAV: influenza A; IBV: influenza B; HRV: human rhinovirus; AdV: adenovirus; PIV: human parainfluenza virus; RSV: respiratory syncytial virus; HCoV-OC43: human coronavirus OC43; HCoV-229E: human coronavirus 229E.


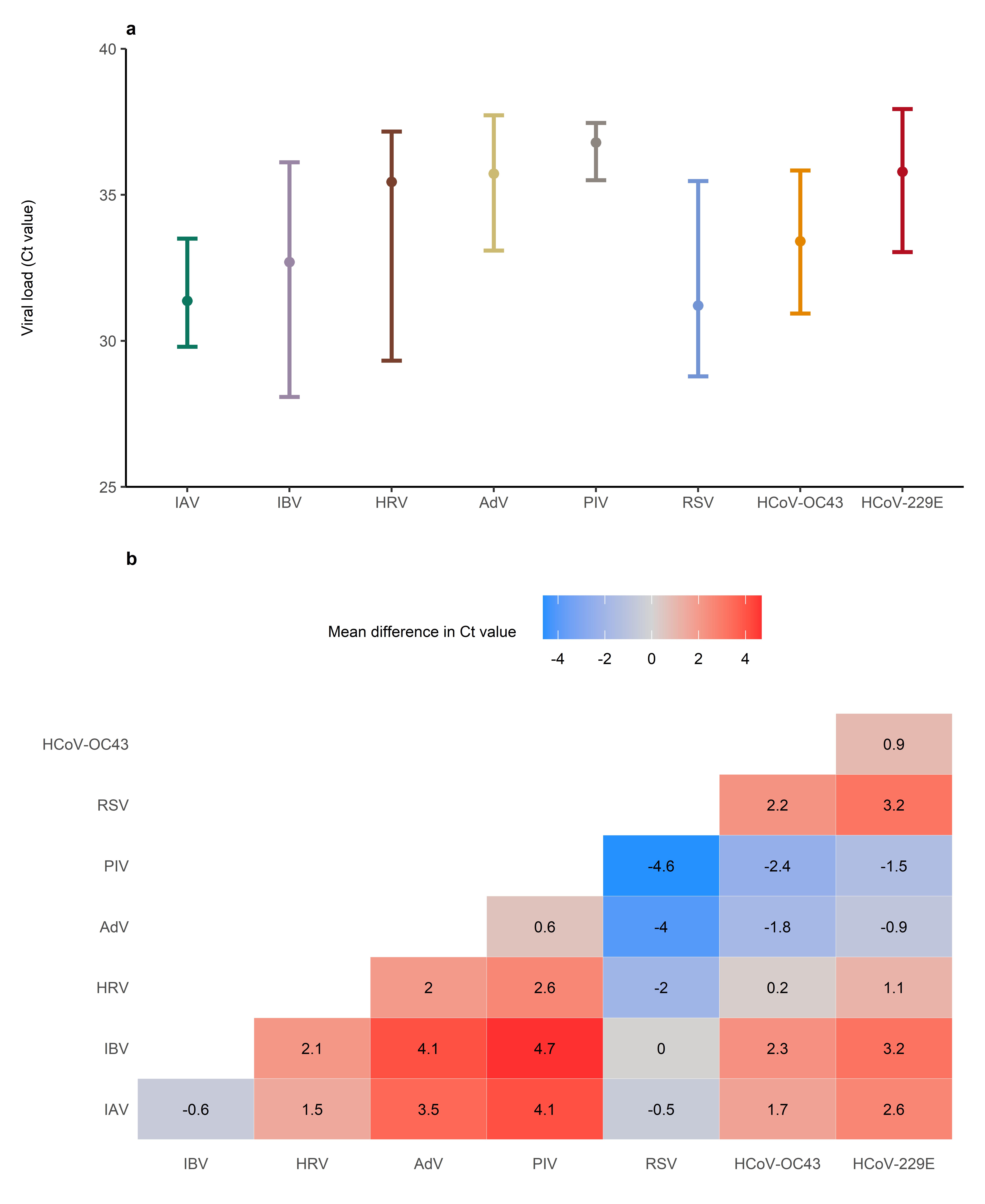


**Supplementary Figure 2: Temporal distribution of positive saliva samples by sex.** Number of positive saliva samples over time by sex. IAV: influenza A; IBV: influenza B; HRV: human rhinovirus; AdV: adenovirus; PIV: human parainfluenza virus; RSV: respiratory syncytial virus; HCoV-OC43: human coronavirus OC43; HCoV-229E: human coronavirus 229E.

**
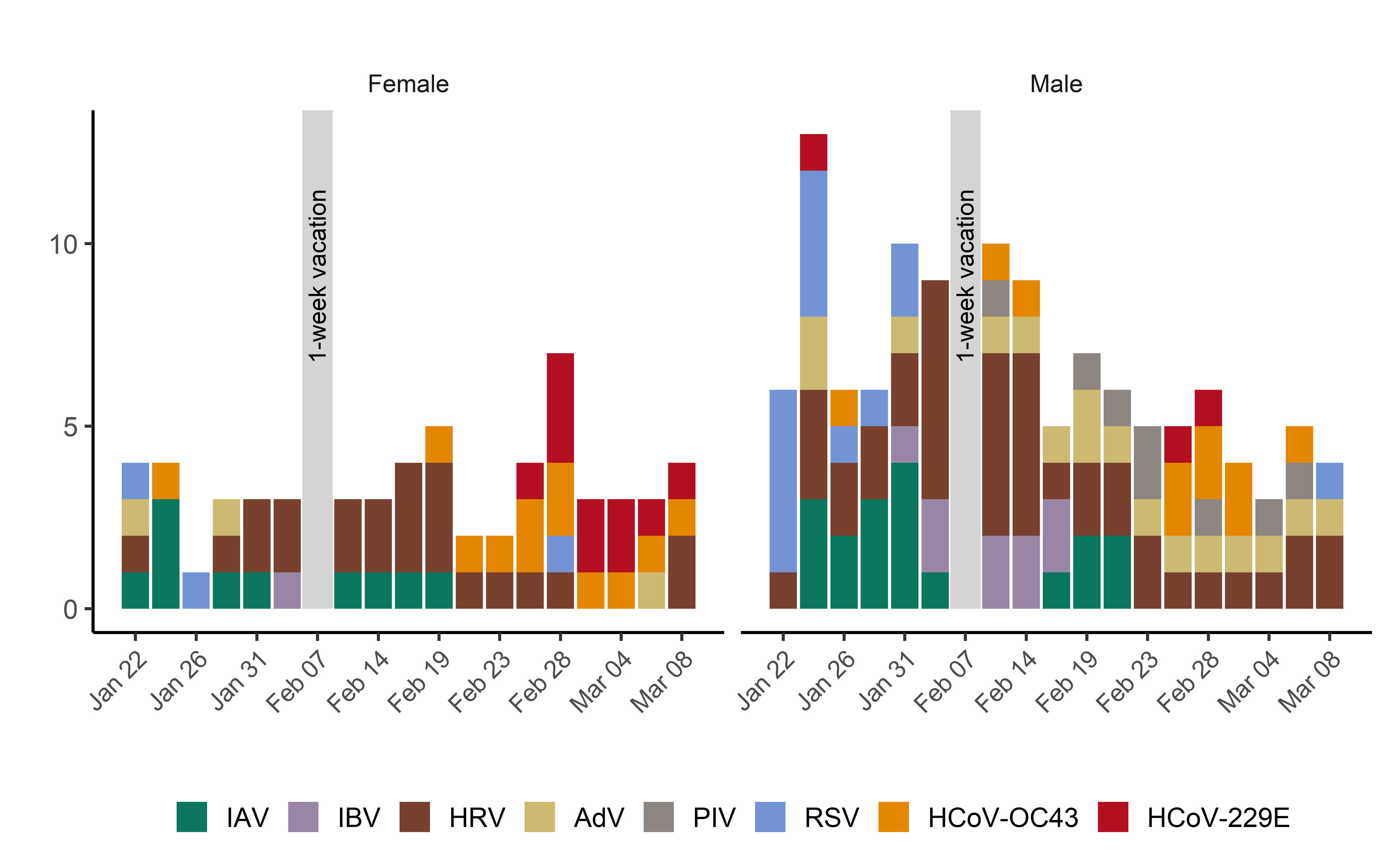
**

**Supplementary Figure 3: Duration of detection of respiratory viruses in saliva.** Observed duration of detection as the number of days testing positive. IAV: influenza A; IBV: influenza B; HRV: human rhinovirus; AdV: adenovirus; PIV: human parainfluenza virus; RSV: respiratory syncytial virus; HCoV-OC43: human coronavirus OC43; HCoV-229E: human coronavirus 229E.


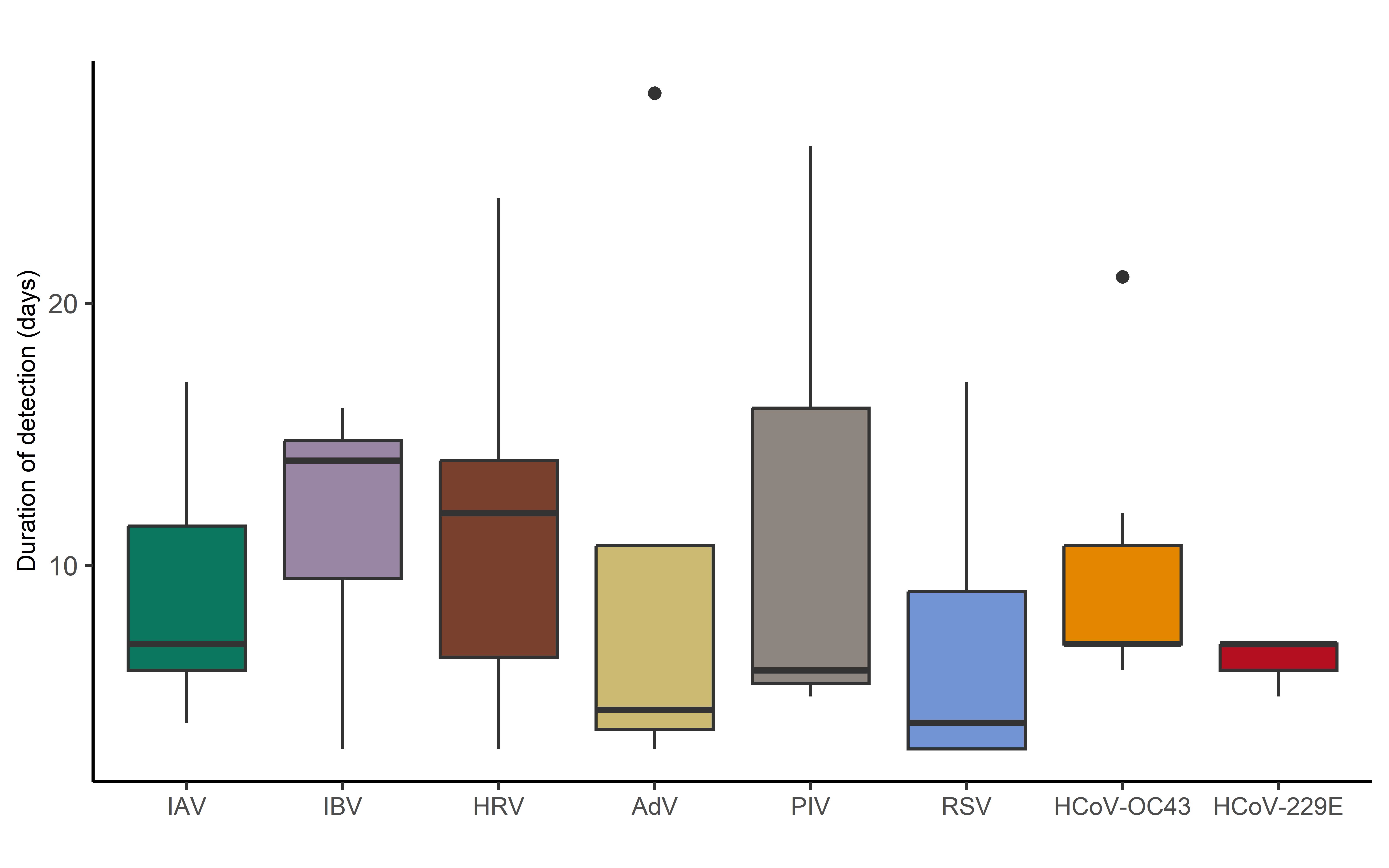


**Supplementary Figure 4:** **Virus-specific duration of detection as survival functions.** Estimated probability of testing positive as function of the number of days since the first positive saliva test result. IAV: influenza A; IBV: influenza B; HRV: human rhinovirus; AdV: adenovirus; PIV: human parainfluenza virus; RSV: respiratory syncytial virus; HCoV-OC43: human coronavirus OC43; HCoV-229E: human coronavirus 229E.


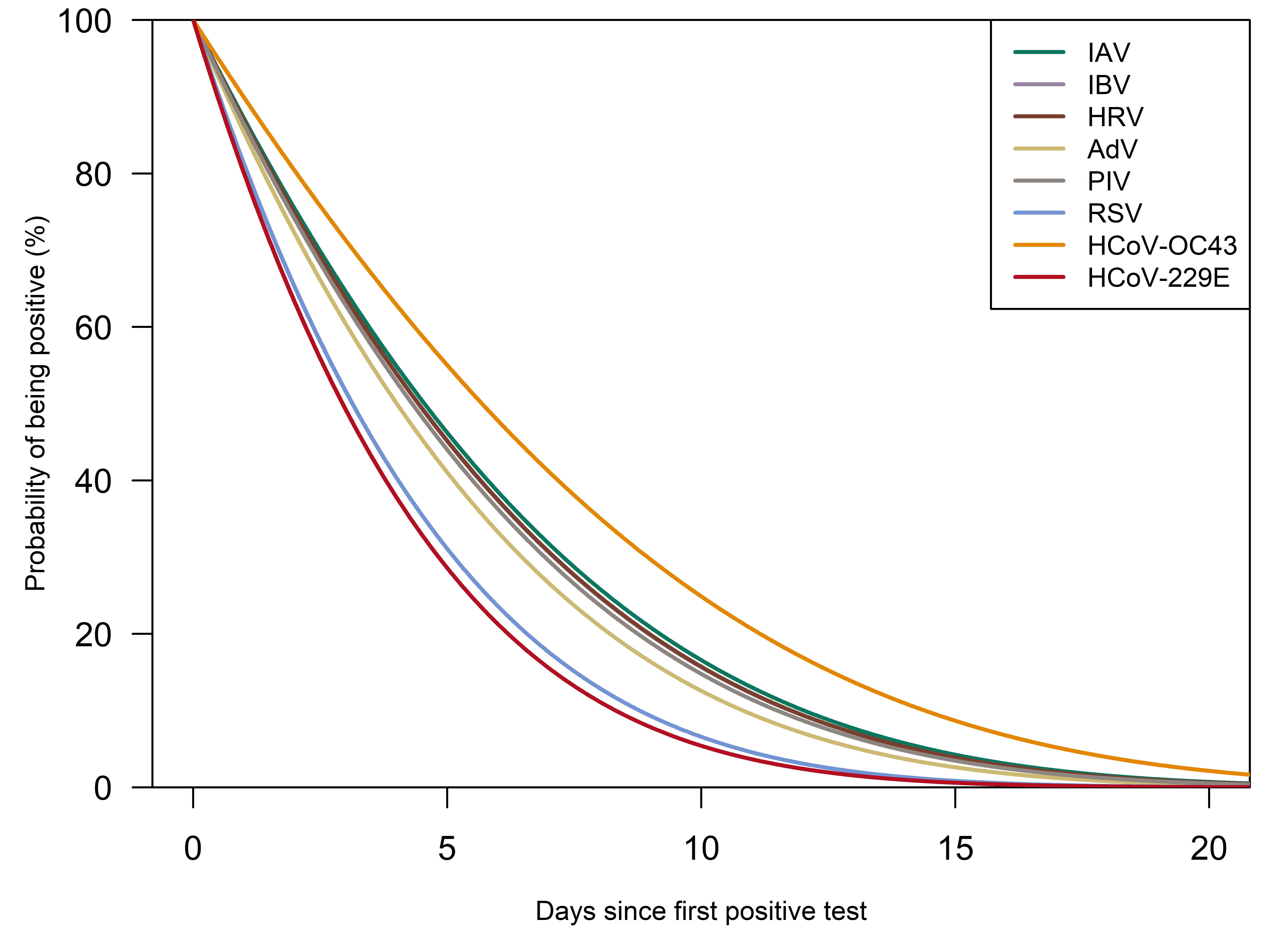


**Supplementary Figure 5: Comparison of the frequencies of viral infections involving school absences.** Risk differences (percentage points) for the proportion of school absences with symptoms of respiratory infections linked to viral infections detected in saliva. IAV: influenza A; IBV: influenza B; HRV: human rhinovirus; AdV: adenovirus; PIV: human parainfluenza virus; RSV: respiratory syncytial virus; HCoV-OC43: human coronavirus OC43; HCoV-229E: human coronavirus 229E.


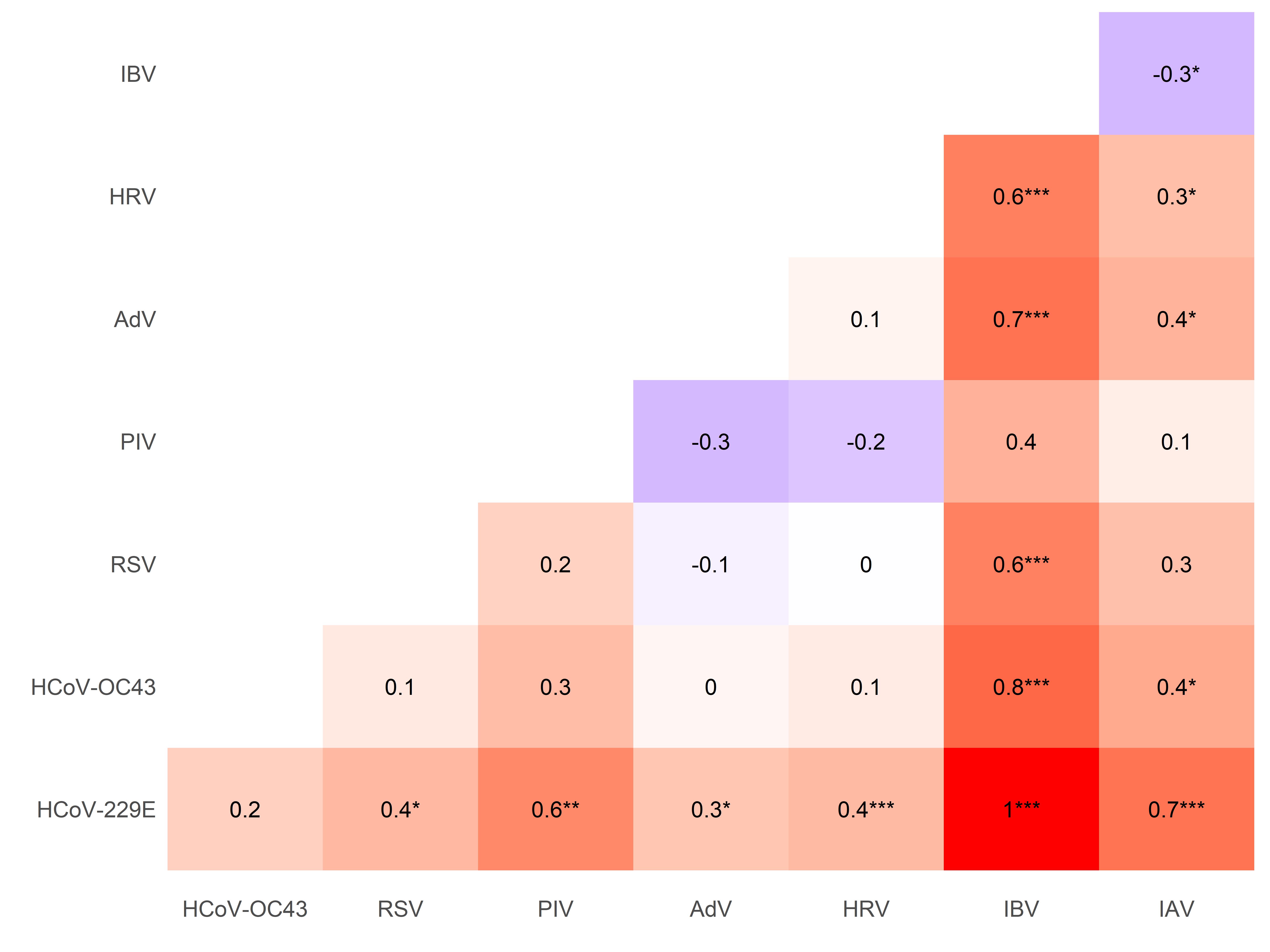


**Supplementary Figure 6: Duration of school absence periods attributed to respiratory viruses.** Observed school absence as the number of days being absent from school due to an illness with symptoms of respiratory infections. IAV: influenza A; IBV: influenza B; HRV: human rhinovirus; AdV: adenovirus; PIV: human parainfluenza virus; RSV: respiratory syncytial virus; HCoV-OC43: human coronavirus OC43; HCoV-229E: human coronavirus 229E.


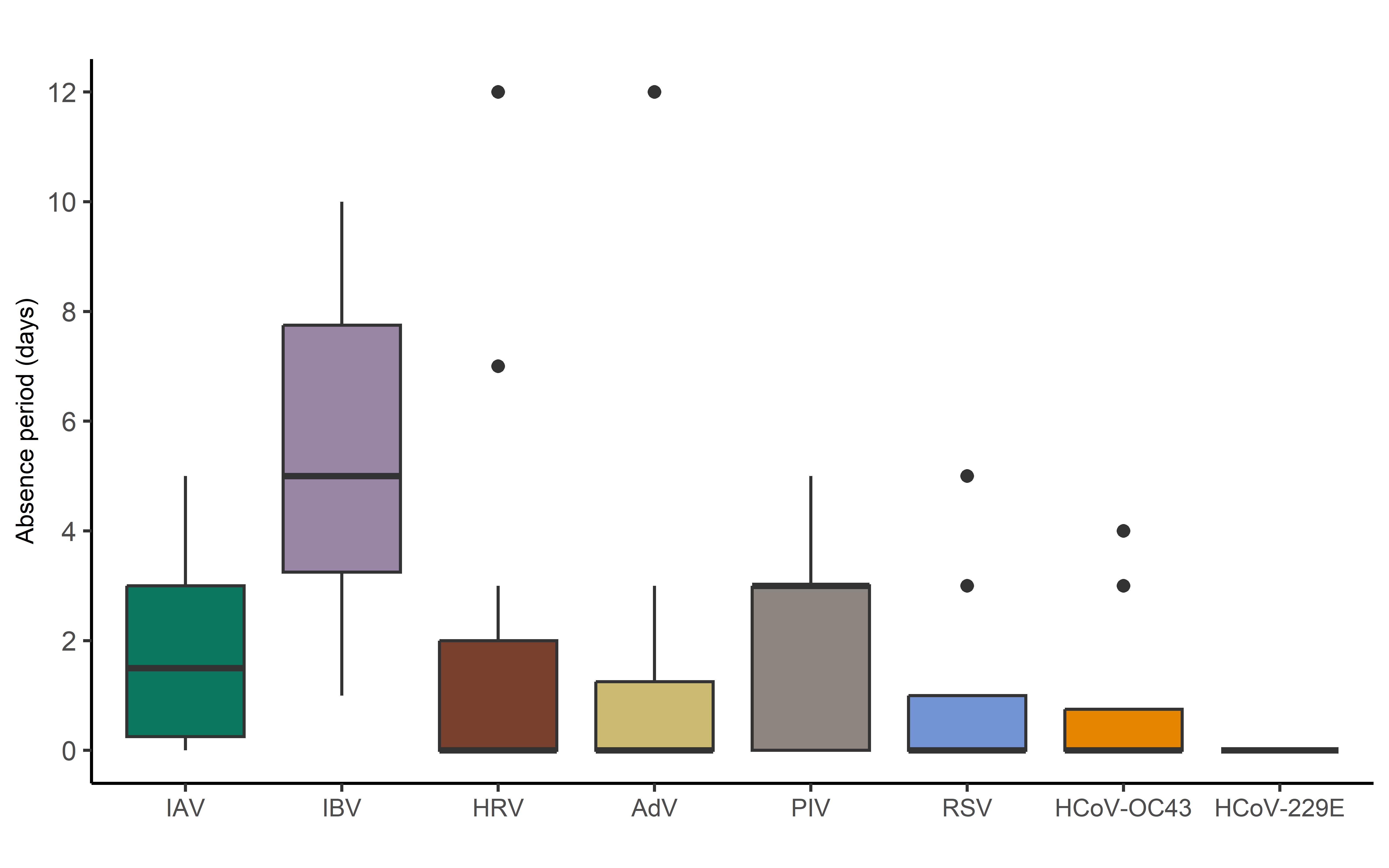


**Supplementary Figure 7:** **Virus-specific absence period as survival functions.** Estimated probability of being absent as function of the number of days since the start of the absence. IAV: influenza A; IBV: influenza B; HRV: human rhinovirus; AdV: adenovirus; PIV: human parainfluenza virus; RSV: respiratory syncytial virus; HCoV-OC43: human coronavirus OC43; HCoV-229E: human coronavirus 229E.


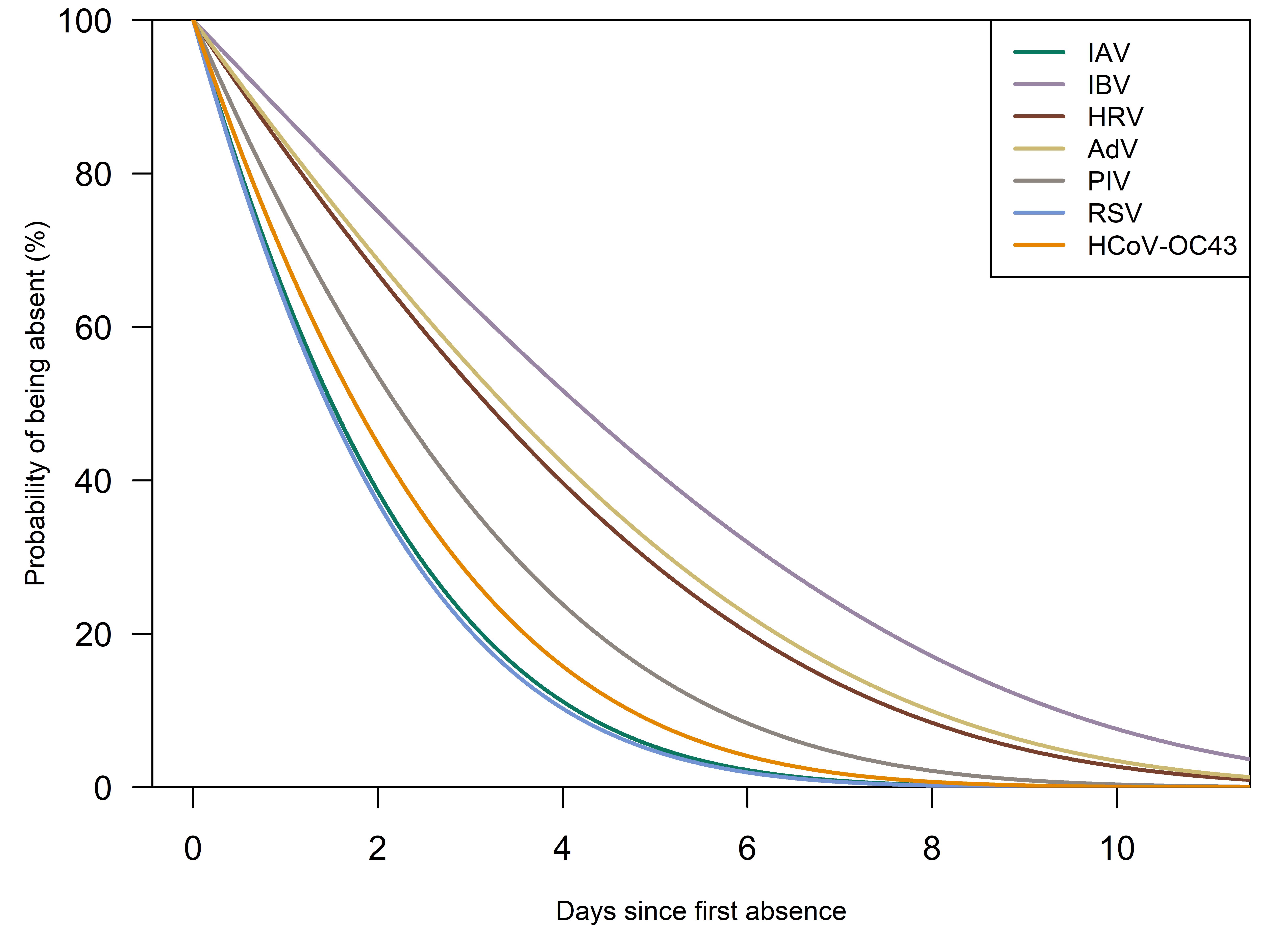


**Supplementary Figure 8: Clustering of respiratory viruses with common symptoms.** Hierarchical cluster analysis showing the cophenetic distance between respiratory viruses based on recorded symptoms. Viruses with common symptoms have a lower distance and are connected closer to the roots of the cluster tree. HCoV-229E not shown because no coinciding absences and symptoms recorded. IAV: influenza A; IBV: influenza B; HRV: human rhinovirus; AdV: adenovirus; PIV: human parainfluenza virus; RSV: respiratory syncytial virus; HCoV-OC43: human coronavirus OC43; HCoV-229E: human coronavirus 229E.


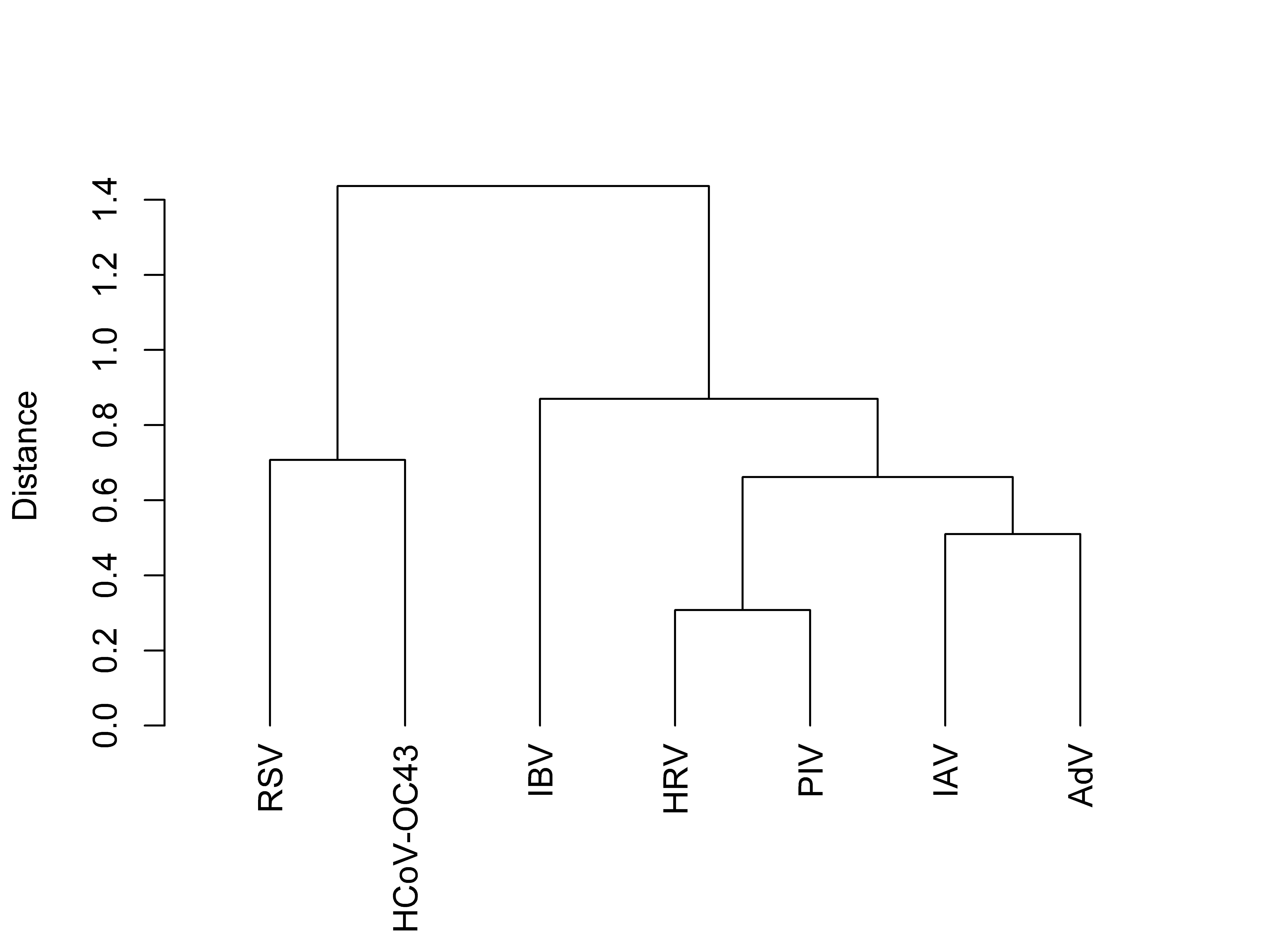


Supplementary Figure 9: Sensitivity analysis for the distribution of symptoms by respiratory virus when excluding absences linked to co-infections. Based on absences attributed to viral infections detected in saliva. Proportion of absences as length of spokes. IAV: influenza A; IBV: influenza B; HRV: human rhinovirus; AdV: adenovirus; PIV: human parainfluenza virus; RSV: respiratory syncytial virus; HCoV-OC43: human coronavirus OC43; HCoV-229E: human coronavirus 229E.


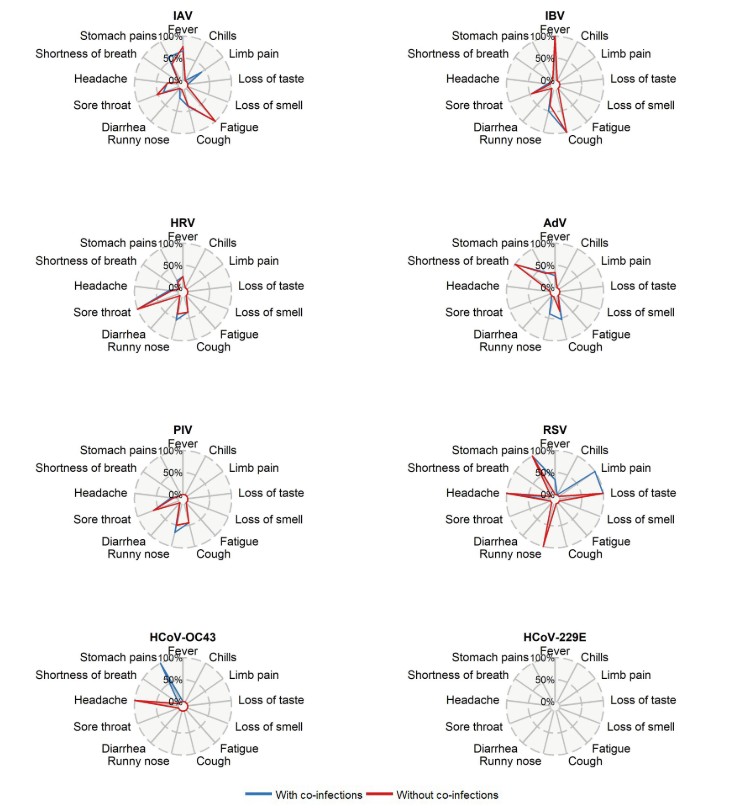


Supplementary Figure 10: Sensitivity analysis for the distribution of symptoms by respiratory virus when linking absences 4, 7 and 10 days before or after a viral infection. Based on absences attributed to viral infections detected in saliva. Proportion of absences as length of spokes. IAV: influenza A; IBV: influenza B; HRV: human rhinovirus; AdV: adenovirus; PIV: human parainfluenza virus; RSV: respiratory syncytial virus; HCoV-OC43: human coronavirus OC43; HCoV-229E: human coronavirus 229E.


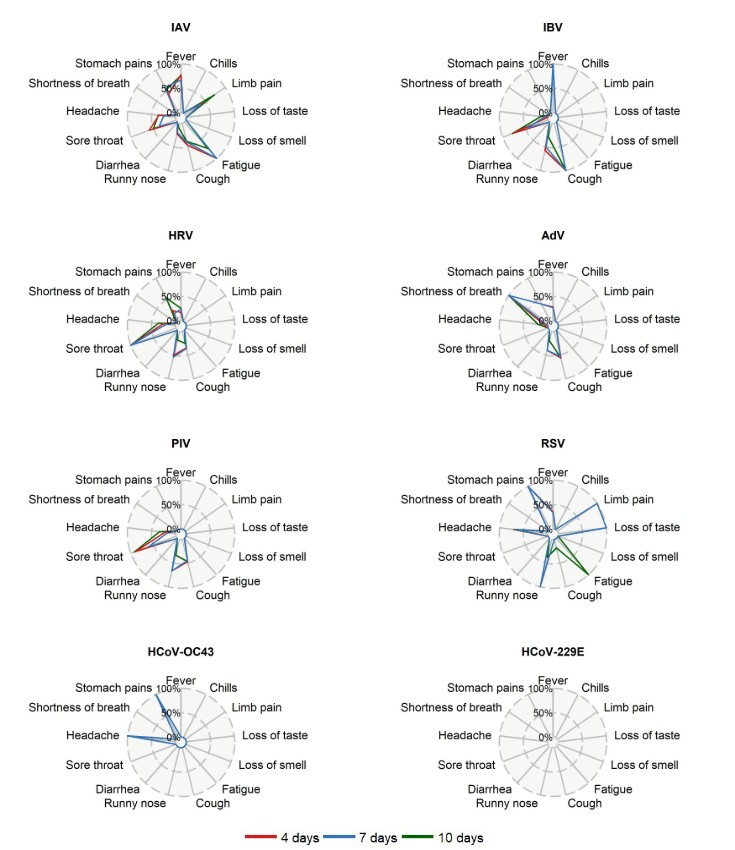

Supplement: Supplementary file 1 — Table S1: Comparison of the duration of detection. Hazard ratio that each pathogen's duration is longer than that of HCoV‐229E (shortest period). IAV: influenza A; IBV: influenza B; HRV: human rhinovirus; AdV: adenovirus; PIV: human parainfluenza virus; RSV: respiratory syncytial virus; HCoV‐OC43: human coronavirus OC43; HCoV‐229E: human coronavirus 229E. Table S2: Comparison of school absence periods. Hazard ratio that each pathogen's period is longer than that of RSV (shortest period). No comparison with HCoV‐229E because no coinciding absences recorded. IAV: influenza A; IBV: influenza B; HRV: human rhinovirus; AdV: adenovirus; PIV: human parainfluenza virus; RSV: respiratory syncytial virus; HCoV‐OC43: human coronavirus OC43; HCoV‐229E: human coronavirus 229E. Figure S1: Comparison of viral loads. (a) Viral loads as the median and interquartile range (IQR) of the lowest Ct values per infection episode. (b) Estimated differences in mean Ct values: p < 0.05 (*), p < 0.01 (**), p < 0.001 (***). IAV: influenza A; IBV: influenza B; HRV: human rhinovirus; AdV: adenovirus; PIV: human parainfluenza virus; RSV: respiratory syncytial virus; HCoV‐OC43: human coronavirus OC43; HCoV‐229E: human coronavirus 229E. Figure S2: Temporal distribution of positive saliva samples by sex. Number of positive saliva samples over time by sex. IAV: influenza A; IBV: influenza B; HRV: human rhinovirus; AdV: adenovirus; PIV: human parainfluenza virus; RSV: respiratory syncytial virus; HCoV‐OC43: human coronavirus OC43; HCoV‐229E: human coronavirus 229E. Figure S3: Duration of detection of respiratory viruses in saliva. Observed duration of detection as the number of days testing positive. IAV: influenza A; IBV: influenza B; HRV: human rhinovirus; AdV: adenovirus; PIV: human parainfluenza virus; RSV: respiratory syncytial virus; HCoV‐OC43: human coronavirus OC43; HCoV‐229E: human coronavirus 229E. Figure S4: Virus‐specific duration of detection as survival functions. Estimated probability of [file IRV-19-e70143-s001.docx]
